# Maternal bile acid profile and subtype analysis of intrahepatic cholestasis of pregnancy

**DOI:** 10.1186/s13023-021-01887-1

**Published:** 2021-06-07

**Authors:** Yong Shao, Siyu Chen, Huan Li, Qin Tang, Di Xu

**Affiliations:** The Department of Obstetrics and Gynecology, The First Affiliated Hospital of Chongqing Medical University, Chongqing, China

**Keywords:** Intrahepatic cholestasis of pregnancy, Bile acid profile, Jaundice, Perinatal outcome

## Abstract

**Background:**

ICP pregnant women have a unique profile of serum bile acid metabolism, thus the early and accurate identification of ICP patients is beneficial to early appropriate treatment and improvement of pregnancy outcomes. In this study, ultra-high performance liquid chromatography-mass spectrometry/mass spectrometry (UPLC-MS/MS) was used to analyze the 15 types of serum bile acid profiles among patients with ICP in third trimester, patients with cholelithiasis, and patients with hepatitis B virus. The ICP diagnostic model established by partial least squares-discriminant analysis (PLS-DA) was used to screen the differential bile acids for clinical subtypes of ICP. 144 cases of ICP patients were involved in this study, and divided into four subgroups according to serum level of TBA, DBIL, and ALT.

**Results:**

(1) The differential serum bile acid profiles of ICP group and normal pregnant women were DCA, TDCA, TCA, GDCA and GLCA. (2) The differential serum bile acid profiles of the ICP1 group (ICP with jaundice) and normal pregnant women were TCDCA, TCA, GCA, GCDCA, TUDCA and GUDCA. (3) The differential serum bile acid profiles of the ICP3 group (Hyperchoicemia of pregnancy) and normal pregnant group was GUDCA, LCA, GLCA, UDCA, TUDCA, CDCA, and TLCA (P < 0.05). (4) The differential serum bile acid profiles of ICP4 group (idiopathic aminotransferase abnormality during pregnancy) and normal pregnant group was UDCA, GUDCA, TUDCA, GCA and GLCA (P < 0.05). (5) The occurrence of meconium-stained amniotic fluid, premature delivery and cesarean section in ICP1 group was significantly higher than normal group, ICP2 group, ICP3 group, and ICP4 group (P < 0.05); The occurrence of meconium-stained amniotic fluid, premature delivery and cesarean section in ICP2 group, ICP3 group, and ICP4 group was significantly higher than normal group (P < 0.05), but no difference was found among ICP2 group, ICP3 group, and ICP4 group (P > 0.05).

**Conclusion:**

Maternal serum bile acid profiles are useful to differentiate the four subtypes of ICP. ICP with jaundice could be an important predictor of adverse pregnancy outcomes of ICP.

**Supplementary Information:**

The online version contains supplementary material available at 10.1186/s13023-021-01887-1.

## Introduction

Intrahepatic cholestasis of pregnancy (ICP) is an idiopathic disease during pregnancy. It is characterized by pruritus and increased levels of total bile acid (TBA) [1]. The main risk of ICP is prone to premature birth, fetal distress, and even unexpected fetal death [2]. Maternal elevated TBA level is currently the most important laboratory indicator for the diagnosis of ICP [3], however, the exclusion of elevated TBA caused by other liver diseases is also necessary. Many studies indicated that the development of ICP is characterized by the disorders in bile acid metabolism, which would finally lead to intrahepatic accumulation of toxic bile acids, and might cause adverse pregnancy outcomes [4]. Therefore, for pregnant women with abnormal liver function, early and accurate identification of ICP is essential, since early intervention of ICP was proven to significantly improve pregnancy outcomes [5].

In this study, ultra-high performance liquid chromatography-mass spectrometry/mass spectrometry (UPLC-MS/MS) was used to analyze the serum bile acid profile among patients with ICP in third trimester, normal pregnant women, patients with cholelithiasis, and patients with hepatitis B virus. The ICP diagnostic model established by partial least squares-discriminant analysis (PLS-DA) was used to screen the differential bile acids for clinical subtype of ICP.

## Methods

### Patients

The 294 cases of study subjects were all from the First Affiliated Hospital of Chongqing Medical University from November 2017 to December 2018, including 50 patients with gallstone disease (including common bile duct stonesand gallbladder stones), 50 patients with hepatitis B virus, 50 cases of normal pregnant women (control group) and 144 cases of pregnant women with ICP. All ICP patients were divided into four subgroups according to maternal serum total bile acid (TBA), direct bilirubin (DBIL), and alanine aminotransferase (ALT), namely the ICP1 group, ICP2 group, ICP3 group and ICP4 group (see Table 1). The ICP1 group was pregnant women with elevated level of TBA, DBIL and ALT; the ICP2 group was pregnant women with elevated level of TBA and ALT but without jaundice (normal DBIL); the ICP3 group (Hyperchoicemia of pregnancy) was pregnant women with elevated level of TBA but normal DBIL and ALT; and the ICP4 group (idiopathic abnormal liver enzymes) was pregnant women with elevated level of ALT but normal TBA and DBIL.

**Table 1:** Subtypes of ICP

Subtype	Cases (n)	TBA (umol/L)	DBIL (umol/L)	ALT (U/L)
ICP1	51	↑	↑↑	↑
ICP2	27	↑	Normal	↑
ICP3	22	↑	Normal	Normal
ICP4	44	Normal	Normal	↑

ICP1 group is pregnant women with jaundice, ICP2 group is pregnant women without jaundice, ICP3 group is pregnant women with hyperbiliary acidemia during pregnancy, ICP4 group is pregnant women with idiopathic liver enzyme abnormalities

The diagnosis of ICP is based on an increase of maternal serum TBA and/or alanine aminotransferase in the second and third trimesters of pregnancy. ICP pregnant women have been excluded from viral hepatitis (hepatitis A, B, C, D, E virus, EB virus, cytomegalovirus, etc.), liver and gallstones, acute fatty liver during pregnancy, preeclampsia, gestational diabetes mellitus, autoimmune liver disease, pregnant women with drug-induced liver injury, and other medical complications; and maternal elevated serum TBA and/or ALT can not be explained by any other disease. All subjects were collected with early morning fasting venous blood before ursodeoxycholic acid (UDCA) treatment (250 mg tid or qid each day) for ICP, and then centrifuged and collected the supernatant with storing in − 80 °C refrigerator for future use. This study was approved by the ethical committee of the First Affiliated Hospital of Chongqing Medical University and complies with the Declaration of Helsinki (as revised in Tokyo 2004). Written informed consent was obtained from all participants.

### Experimental instrument

Waters Xevo TQD IVD tandem mass spectrometry, Acquity UPLC I-Class ultra-performance liquid chromatography, Masslynx 4.1 workstation, Waters ACQUITY UPLC BEH C18 chromatographic column, etc. were purchased from Waters Corporation in the United States.

### Experiment reagent

Glycholithocholic acid (GLCA) standards were purchased from Toronto Research Chemicals; Cholic acid (CA), lithocholic acid (LCA), deoxycholic acid (DCA), ursodeoxycholic acid (UDCA), chenodeoxycholic acid (CDCA), taurocholic acid (TCA), glychocholic acid (GCA), taurolithocholic acid (TLCA), taurodeoxycholic acid (TDCA), tauroursodeoxycholic acid (TUDCA), taurochenodeoxycholic acid (TCDCA), glychodeoxycholic acid (GDCA), glychochenodeoxycholic acid (GCDCA), glychoursodeoxycholic acid (GUDCA) standards, and isotope-labeled internal standard deuterocholic acid (d4-CA), deuterodeoxycholic acid (d4-DCA), deuteroglychocholic acid (d4-GCA), deuterated ursodeoxycholic acid (d4-UDCA), and deuterated lithocholic acid (d4-LCA) were purchased from Sigma Aldrich; Chromatographic pure acetonitrile and chromatographic pure methanol were purchased from Merck, Germany; Chromatographic grade formic acid was purchased from Shanghai Aladdin.

### Chromatographic and mass spectrometric conditions

Chromatographic column: Waters ACQUITY UPLC BEH C18; Mobile phase: 0.1% formic acid solution in phase A (volume ratio), 0.1% formic acid—acetonitrile: methanol (3:1, v:v) solution in phase B; Flow rate: 0.4 mL/min; Column temperature: 45 °C; Gradient elution procedure: 0 ~ 2 min, 35% ~ 43%, B; 2.0 ~ 3.5 min, 43% ~ 46%, B; 3.5 ~ 5.0 min, 46% ~ 59%, B; 5.0 ~ 7.0 min, 59%, B; 7.0 ~ 8.7 min, 59% ~ 66%, B; 8.7 ~ 10.7 min, 66% ~ 98%, B; 10.7 ~ 11.3 min, 35%, B.

Ion source: electrospray ion source (ESI); Scanning mode: anion scanning; Detection mode: multi-response monitoring (MRM) mode; Capillary voltage: 3.0kv; Ion source temperature: 150 °C; Desolvent temperature: 400 °C; Desolvent gas flow rate: 800 L/h; Cone hole gas flow rate: 50 L/h. Time segments were used to improve the response value of the objects to be measured, and the mass spectrum parameters of 15 bile acids were shown in Table 2.

**Table 2:** The mass spectrometric parameters of 15 kinds of bile acids

Name	Parent ion (m/z)	Product ion (m/z)	Dwell time (s)	Cone voltage (V)	Collision energy (eV)
LCA DCA	375.3 391.3	375.3 391.3	0.18 0.18	− 95 − 84	10 10
CDCA CA	391.3 407.3	391.3 407.3	0.25 0.18	− 84 − 80	10 10
UDCA	391.3	391.3	0.20	− 84	10
GLCA GDCA GCDCA GCA	432.3 448.3 448.3 464.3	74.0 74.0 74.0 74.0	0.16 0.18 0.18 0.17	− 64 − 74 − 74 − 76	36 40 40 42
GUDCA	448.3	74.0	0.16	− 74	40
TLCA	482.3	80.0	0.20	− 88	60
TDCA	498.3	80.0	0.23	− 92	62
TCDCA	498.3	80.0	0.23	− 92	62
TCA	514.3	80.0	0.16	− 90	67
TUDCA	498.3	80.0	0.17	− 92	62

In this study, the low, medium and high quality control are standard products that are added to the blank serum. The internal standard is in matrix. The standard curve is made using blank serum instead of mobile phase as internal standard and the matrix effect is eliminated. Four times charcoal-stripped plasma (Na^+^ EDTA as anticoagulant) was further striped in our laboratory by mixing 0.1 g of active charcoal with approximately 10 mL of plasma and gently shaking at 4 °C for 24 h. The mixture was first centrifuged at 1650×*g* for 15 min and the supernatant was centrifuged at14,000×*g* for 30 min. The resulting supernatant was filtered by gravity through a 25-wm Whatman folded filter and than blank serum was collected for future use.

### Statistical analysis

The SIMCA-P 13.0 software (Umetrics, Sweden) is used to perform PLS-DA for screening of bile acid spectrum and differential bile acid spectrum. The analytic results are expressed in two-dimensional and three-dimensional score plots. Other statistical analysis are performed using SPSS 21.0 software (IBM Corporation, USA). The data are tested for normality using the Kolmogorov-Smirnon test. Most of the measurement data in this study are non-normal distribution, which is expressed by Median (Q1, Q3). Kruskal–Wallis H rank sum test is used for measurement data, and Mann–Whitney U test modified by Bonferroni is used for pairwise comparison between groups. The test of the count data and the pairwise comparison between the groups are selected according to the characteristics of the data using Pearson chi-square test, continuously corrected chi-square test or Fisher exact probability method. Test level a = 0.05; Pairwise comparisons between multiple groups use the test level corrected by Bonferroni.

## Results

### Methodological evaluation and quality control

A good linear relationship of 15 kinds of bile acids is shown in the linear range of 1.0 to 60,000 nmol/L, and the linear correlation coefficients (R2) are all over 0.993; the limit of quantification of the target is from 1.0 to 10.0 nmol/L. If a sample is more concentrated than the last point on the standard curve, the sample was diluted and than tested. The low, medium and high concentration recovery rates ranged from 95.5 to 114.1%, the intra-day and inter-day RSDs were less than 11.4%, and the quality control coefficients of variation of 15 bile acids were less than15%.

### Analysis of serum bile acid profile in ICP group, normal pregnant women group, cholelithiasis group and hepatitis B virus group

**Fig. 1.**
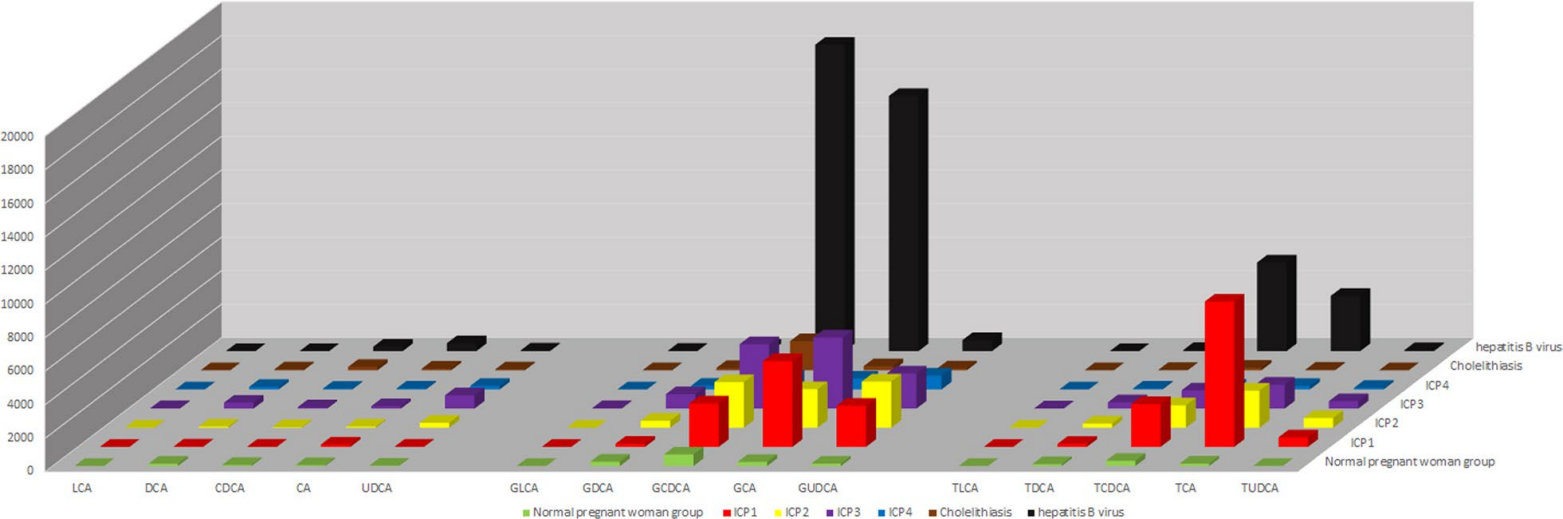
15 kinds of serum bile acids profiles analysis in different groups. *Note*: data unit is nmol/L

The serum bile acid profile of the ICP group was different from normal pregnant group, the cholelithiasis group and the hepatitis B virus group. The bile acid spectrum characteristics of the ICP1 group, ICP2 group, and ICP3 group were similar, and the serum concentrations of GCDCA, GCA, GLCA, TDCA, TCA, GUDCA, and TUDCA were significantly higher than normal pregnant group (P < 0.008). The serum bile acid profile of ICP4 group was different from normal pregnant group, ICP1 group, ICP2 group, ICP3 group, cholelithiasis group and hepatitis B virus group, and the serum concentrations of UDCA, GUDCA, TUDCA, GCA and GLCA in ICP4 group were significantly higher than normal pregnant group (P < 0.01) (see Fig. 1, Table 3).

**Table 3.** 15 Kinds of serum bile acids in different groups

	Normal pregnant woman group (n = 50)	ICP1 (n = 51)	ICP2 (n = 27)	ICP3 (n = 22)	ICP4 (n = 44)	Cholelithiasis (n = 50)	hepatitis B virus (n = 50)	H value	P value
LCA	6.78 (5.15, 12.87)	3.81 (0.37, 12.22)^a^	9.73 (1.59, 16.04)	27.97 (6.19, 65.14)^a,b,c,d^	7.60 (1.74, 16.78)	7.46 (4.61, 18.13)^b^	3.38 (0.00, 11.91)	26.389	0.000
UDCA	26.56 (12.91, 63.12)	16.40 (5.99, 2156.61)	299.49 (16.25, 959.76)^a^	791.55 (343.89, 1685.69)^a,c,d^	211.79 (41.88, 611.20)^a,c,d^	57.35 (10.75, 145.08)	39.90 (8.51, 137.31)	39.197	0.000
CDCA	63.78 (22.29, 127.39)	14.37 (7.58, 55.29)^a^	36.55 (11.71, 85.63)	83.95 (37.14, 344.09)^b,c,d^	41.46 (11.29, 79.14)^c,d^	201.44 (70.61, 691.10)^a,b^	269.75 (83.64, 682.13)^a,b^	91.967	0.000
DCA	134.22 (61.01, 189.83)	23.89 (4.20, 86.86)^a^	69.51 (12.71, 148.48)	354.69 (96.47, 699.47)^b,d^	154.29 (71.14, 283.26)^b,d^	57.75 (6.05, 375.86)	6.00 (2.04, 161.40)^a^	40.844	0.000
CA	55.06 (30.96, 133.01)	140.71 (38.58, 543.40)	67.84 (29.19, 119.43)	146.77 (60.03, 648.8)	56.55 (25.88, 196.19)^d^	101.27 (35.50, 304.24)	470.85 (114.04, 1689.49)^a,b^	61.819	0.000
GLCA	3.39 (0.00, 6.67)	4.46 (2.27, 15.82)^a^	5.75 (2.92, 27.64)^a^	28.88 (7.07, 65.77)^a,b,c^	6.28 (3.24, 13.80)^a,d^	5.27 (3.91, 15.48)^a^	19.81 (5.78, 39.46)^a,b^	24.321	0.000
GUDCA	141.22 (77.47, 223.13)	2453.22 (632.41, 4416.73)^a^	2753.28 (1263.88, 5018.57)^a^	2092.63 (1277.99, 3306.3)^a,c,d^	824.15 (364.58, 1524.56)^a,b,c^	115.62 (64.37, 341.49)^b^	659.29 (314.72, 2030.89)^a,b^	150.749	0.000
GCDCA	689.64 (405.26, 1155.35)	2587.20 (1447.06, 4949.62)^a^	2716.70 (1616.13, 4433.8)^a^	3834.13 (2001.43, 6069.68)^a,c,d^	722.24 (385.83, 994.84)^b,c,d^	1734.37 (958.24, 4784.75)^a^	18, 254.50 (6526.70, 60, 229.00)^a,b^	159.991	0.000
GDCA	259.82 (125.04, 431.66)	185.93 (62.06, 504.23)	416.33 (123.87, 1123.29)	861.1 (497.66, 2153.76)^a,b,c,d^	222.82 (93.66, 427.65)	137.46 (43.21, 333.13)	270.39 (22.89, 824.99)	39.306	0.000
GCA	245.02 (160.14, 405.87)	5107.28 (2337.71, 10, 380.40)^a^	2297.14 (1520.24, 6281.02)^a^	4235.73 (2052.99, 9929.26)^a,c,d^	647.49 (539.83, 934.85)^a,b,c^	243.94 (97.07, 1302.62)^b^	15, 199.35 (3820.78, 62, 330.67)^a,b^	54.292	0.000
TLCA	3.25 (0.00, 6.09)	5.58 (4.05, 14.36)^a^	7.23 (3.42, 17.50)^a^	14.72 (4.06, 28.76)^a,c,d^	2.88 (1.65, 4.03)^b,c^	4.33 (2.84, 6.67)	4.26 (0.32, 12.07)	128.749	0.000
TUDCA	9.09 (3.72, 16.92)	565.99 (27.01, 1972.32)^a^	581.77 (53.22, 984.79)^a^	441.97 (144.24, 1222.01)^a,c,d^	96.89 (37.62, 164.88)^a,c^	5.25 (3.34, 14.94)^b^	63.8 (13.07, 149.13)^a,b^	141.125	0.000
TDCA	111.11 (54.11, 187.14)	182.08 (81.71, 443.78)	248.21 (73.67, 585.44)	375.06 (72.56, 507.48)^a,c,d^	59.24 (27.70, 124.67)^b,c^	20.98 (5.00, 80.12)^a,b^	72.91 (10.09, 188.84)^b^	47.217	0.000
TCDCA	313.07 (148.56, 547.40)	2546.27 (1213.19, 6120.77)^a^	1333.51 (845.08, 3308.70)^a^	1082.66 (470.28, 1458.14)^a,b,c,d^	168.34 (110.16, 402.18)^b,d^	156.72 (83.72, 1183.87)^b^	5285.51 (1169.15, 16, 671.87)^a^	123.417	0.000
TCA	143.90 (91.89, 319.64)	8656.79 (2542.60, 20, 058.10)^a^	2210.20 (866.51, 5769.00)^a,b^	1425.13 (830.08, 4644.10)^a,b,c^	209.71 (129.25, 434.93)^b,c,d^	42.78 (18.52, 360.43)^a,b^	3294.19 (667.89, 13, 783.69)^a^	116.092	0.000

The data in the table is expressed by Median (Q1, Q3). Data unit is nmol/L. Pairwise comparisons between groups were performed using Bonferroni correction

^a^Compared with the normal pregnant women group, P < 0.008

^b^Compared with the ICP1 group, P < 0.01

^c^Compared with gallstone group P < 0.025

^d^Compared with the hepatitis B virus group, P < 0.025

### Analysis of serum bile acid profile in each subgroup of ICP

Based on the data of 15 known serum bile acids by mass spectrometry, PLS-DA was used to analyze the serum bile acid profiles of each subgroup of ICP and normal pregnant women. The parameters of the constructed PLS-DA model variables R2Y represents the explanatory ability of the model, and Q2 represents the ability of the model to predict new data. The VIP value of various cholic acids is the contribution value of the subgroups on the PLS-DA score chart. Cholic acid with a VIP value > 1 could be used as the significantly differential cholic acids between two comparision groups.

**Fig. 2:**
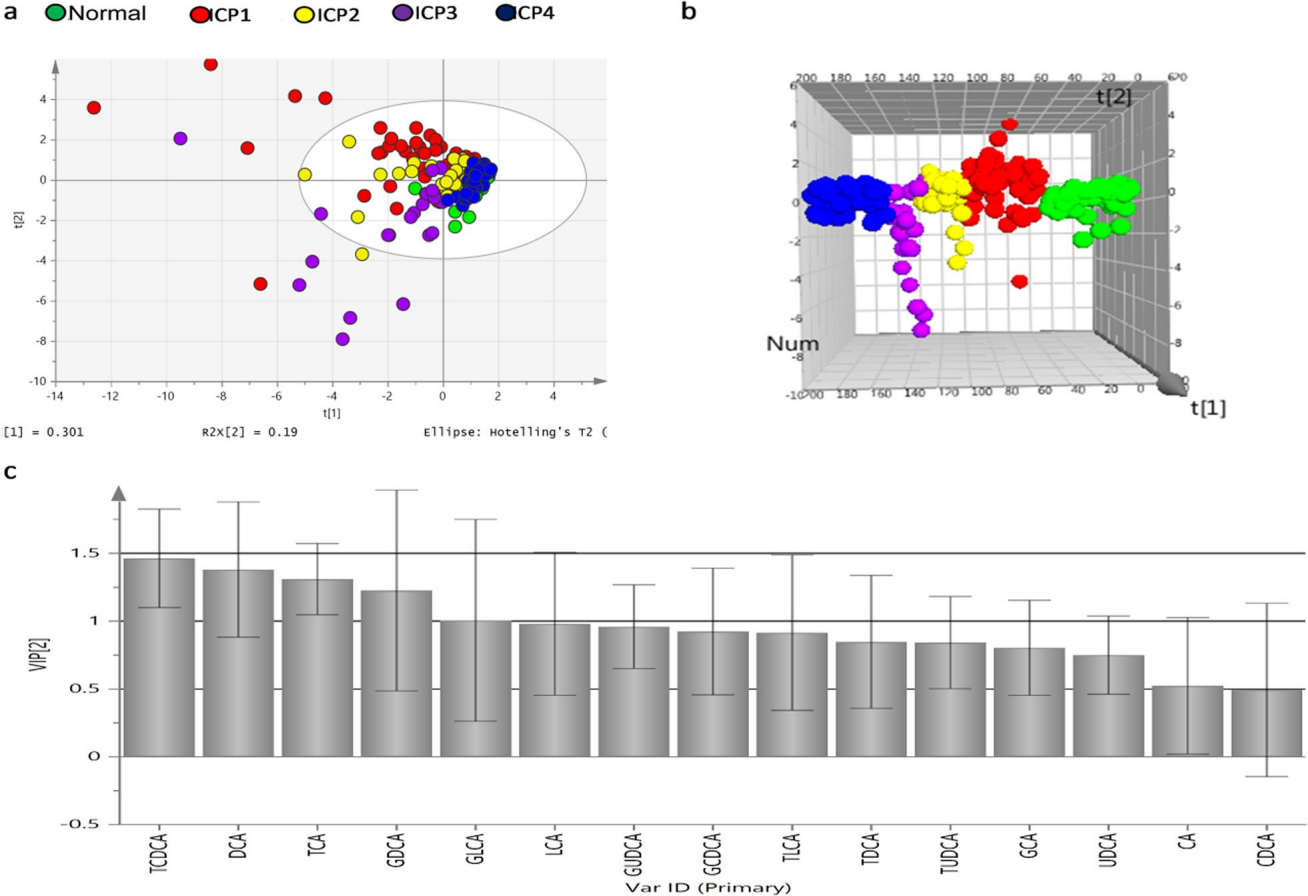
PLS-DA analysis of serum bile acid in ICP subgroups and normal pregnant women. *Note*: **a** Two-dimensional score chart; **b** three-dimensional score chart; **c** VIP values

PLS-DA models were established with ICP subgroups and normal pregnant women group (R2Y = 0.156, Q2 = 0.126). The ICP subgroups and normal group can be differentiated very well based on the data of bile acid profile under the three-dimensional score chart than two-dimensional score chart (see Fig. 2a, b). Figure 2c and Table 4 showed the VIP values of differential cholic acid for the five groups (see Fig. 2c and Table 4). The differential bile acids in the ICP subgroups (ICP1 group, ICP2 group, ICP3 group and ICP4 group) and normal pregnant women group were TCDCA, DCA, TCA, GDCA and GLCA.

**Table 4:** Analysis of serum differential bile acid profiles of group ICP1, group ICP2, group ICP3, group ICP4 and normal pregnant women

Differential bile acid	VIP value	Comparison of concentrations in five groups
TCDCA	1.463	ICP1 > ICP2 > ICP3 > normal pregnant > ICP4
DCA	1.380	ICP3 > ICP4 > normal pregnant > ICP2 > ICP1
TCA	1.309	ICP1 > ICP2 > ICP3 > ICP4 > normal pregnant
GDCA	1.226	ICP3 > ICP2 > ICP1 > normal pregnant > ICP4
GLCA	1.006	ICP3 > ICP2 > ICP1 > ICP4 > normal pregnant

### Analysis of serum bile acid profile of ICP1 group (ICP with jaundice)

**Fig. 3:**
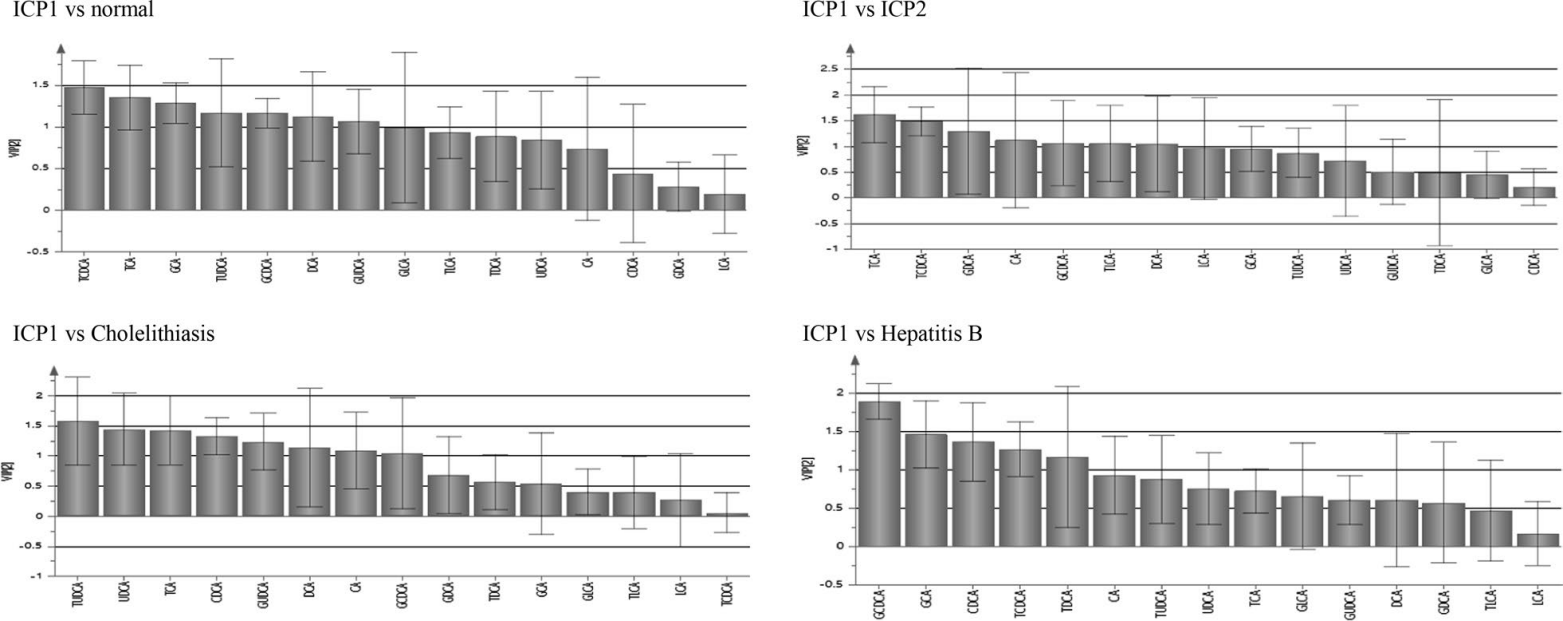
PLS-DA analysis of serum bile acid profiles VIP value between groups (ICP1 vs normal group, ICP2, Cholelithiasis and Hepatitis B)

The differential bile acids between ICP1 group and normal pregnant group were TCDCA, GCDCA, TCA, GCA, DCA, TUDCA and GUDCA, and the concentration of these bile acids in ICP1 group were significantly higher than normal pregnant group (P < 0.05). The differential bile acids between ICP1 group and ICP2 group were TCA, TCDCA, GDCA, CA, GCDCA, TLCA and DCA, and the concentration of TCA, TDCA, CA and GCDCA in ICP1 group were higher than ICP2 group (P < 0.05), while the concentration of GDCA, TLCA and DCA in ICP1 group were lower than ICP2 group (P < 0.05). The differential bile acids between ICP1 group and cholelithiasis group were TUDCA, UDCA, TCA, CDCA, GUDCA, DCA, CA, and GCDCA, and the concentration of TUDCA, UDCA, TCA, GUDCA, and CA in ICP1 group were higher than cholelithiasis group (P < 0.05), while the concentration of DCA and GCDCA were lower than cholelithiasis group (P < 0.05). The differential bile acids between ICP1 group and hepatitis B group were GCDCA, GCA, CDCA, TCDCA, and TDCA, and the concentration of TDCA in ICP1 group was higher than hepatitis B group, while the concentration of GCDCA, GCA, CDCA, and TDCA were lower than hepatitis B group (P < 0.05) (see Fig. 3, Table 5).

**Table5:** Analysis of Bile acids profiles between ICP1 and other groups

Comparison groups	Bile acids profile					Comparison results of bile acids concentration between groups
	CA GCA TCA	CDCA GCDCA TCDCA	DCA GDCA TDCA	LCA GLCA TLCA	UDCA GUDCA TUDCA	
ICP1 vs normal group	GCA TCA	GCDCA TCDCA	DCA		GUDCA TUDCA	ICP1 > normal group
ICP1 vs ICP2	CA TCA	GCDCA TCDCA				ICP1 > ICP2
		DCA	GDCA	TLCA		ICP1 < ICP2
ICP1 vs Cholelithiasis	CA TCA				UDCA GUDCA TUDCA	ICP1 > Cholelithiasis
		CDCA GCDCA	DCA			ICP1 < Cholelithiasis
ICP1 vs Hepatitis B			TDCA			ICP1 > Hepatitis B
	GCA	CDCA GCDCA TCDCA				ICP1 < Hepatitis B

### Analysis of serum bile acid profile in ICP3 group (Hyperchoicemia of pregnancy)

**Fig. 4:**
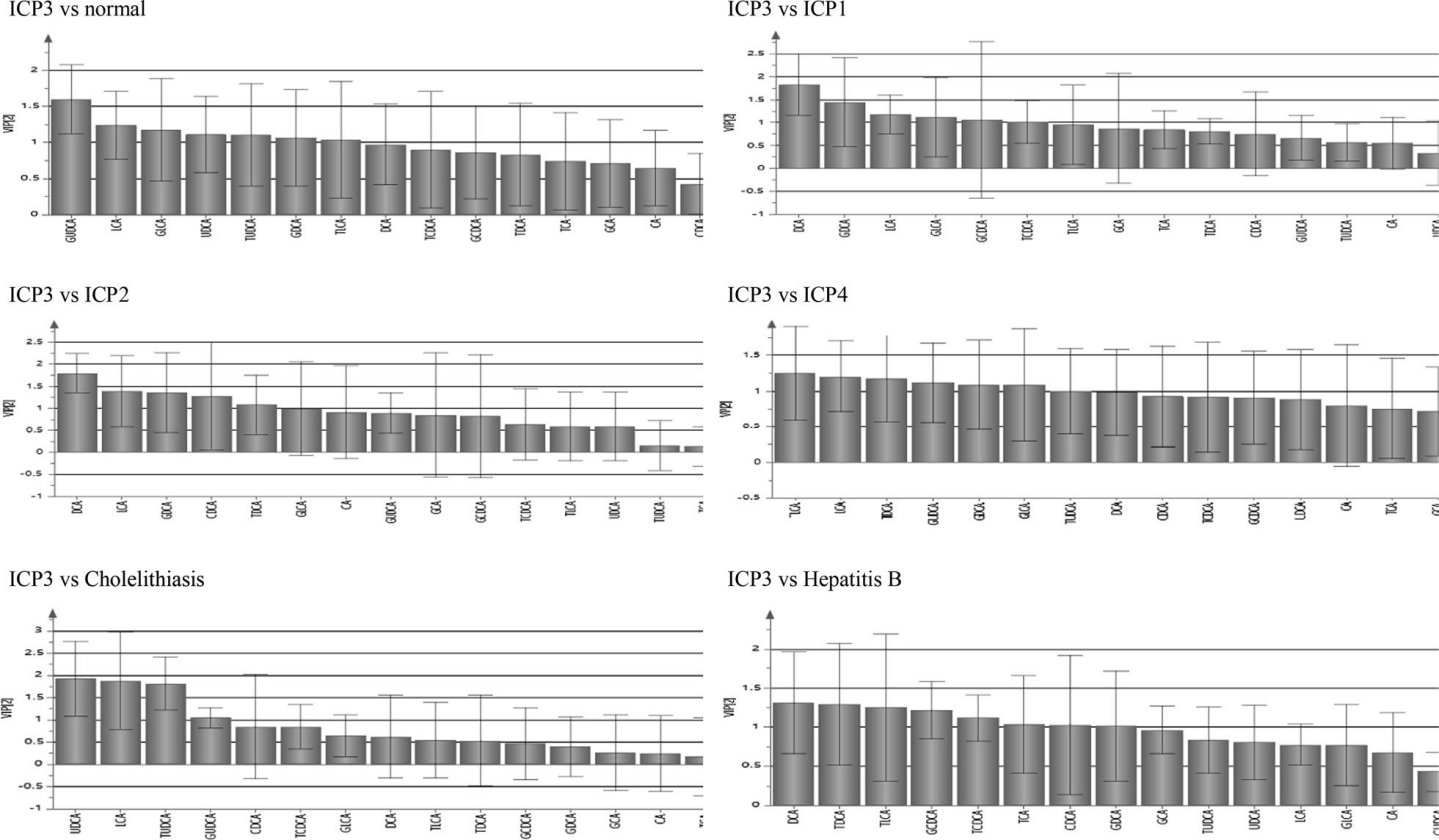
PLS-DA analysis of serum bile acid profiles VIP value between groups (ICP3 vs normal group, ICP1, ICP2, ICP4, Cholelithiasis and Hepatitis B)

The differential bile acids between ICP3 group and normal pregnant group were GUDCA, LCA, GLCA, UDCA, TUDCA, CDCA and TLCA, and the concentration of these bile acids in ICP3 group were higher than normal pregnant group (P < 0.05). The differential bile acids between ICP3 group and ICP1 group were DCA, GDCA, LCA, GLCA, GCDCA, and TDCCA, and the concentration of DCA, GDCA, LCA, GLCA, and GCDCA in ICP3 group were higher than ICP1 group (P < 0.05), while the concentration of TDCA were lower than ICP1 group (P < 0.05). The differential bile acids between ICP3 group and ICP2 group were DCA, GDCA, TDCA, CDCA and LCA, and the concentration of these bile acids in ICP3 group were higher than ICP2 group (P < 0.05). The differential bile acids between ICP3 group and cholelithiasis group were UDCA, GUDCA, TUDCA and LCA, and the concentration of these bile acids in ICP3 group were higher than cholelithiasis group (P < 0.05). The differential bile acids between ICP3 group and hepatitis B group were DCA, TDCA, TLCA, GCDCA, TCDCA, TCA, CDCA, and GDCA, and the concentration of DCA, GDCA, TDCA, and TLCA in ICP3 group were higher than hepatitis B group, while the concentration of TCA, CDCA, GCDCA, TDCA was lower than hepatitis B group (P < 0.05) (see Fig. 4, Table 6).

**Table6:** Analysis of Bile acids profiles between ICP3 and other groups

Comparison groups	Bile acids profile					Comparison results of bile acids concentration between groups
	CA GCA TCA	CDCA GCDCA TCDCA	DCA GDCA TDCA	LCA GLCA TLCA	UDCA GUDCA TUDCA	
ICP3 vs normal group		CDCA		LCA GLCA TLCA	UDCA GUDCA TUDCA	ICP3 > normal group
ICP3 vs ICP1		GCDCA	DCA GDCA	LCA GLCA		ICP3 > ICP1
		TCDCA				ICP3 < ICP1
ICP3 vs ICP2		CDCA	DCA GDCA TDCA	LCA		ICP3 > ICP2
ICP3 vs Cholelithiasis				LCA	UDCA GUDCA TUDCA	ICP3 > Cholelithiasis
ICP3 vs Hepatitis B			DCA GDCA TDCA	TLCA		ICP3 > Hepatitis B
	TCA	CDCA GCDCA TCDCA				ICP3 < Hepatitis B

### Analysis of serum bile acid profile in ICP4 group (idiopathic aminotransferase abnormality during pregnancy)

**Fig. 5:**
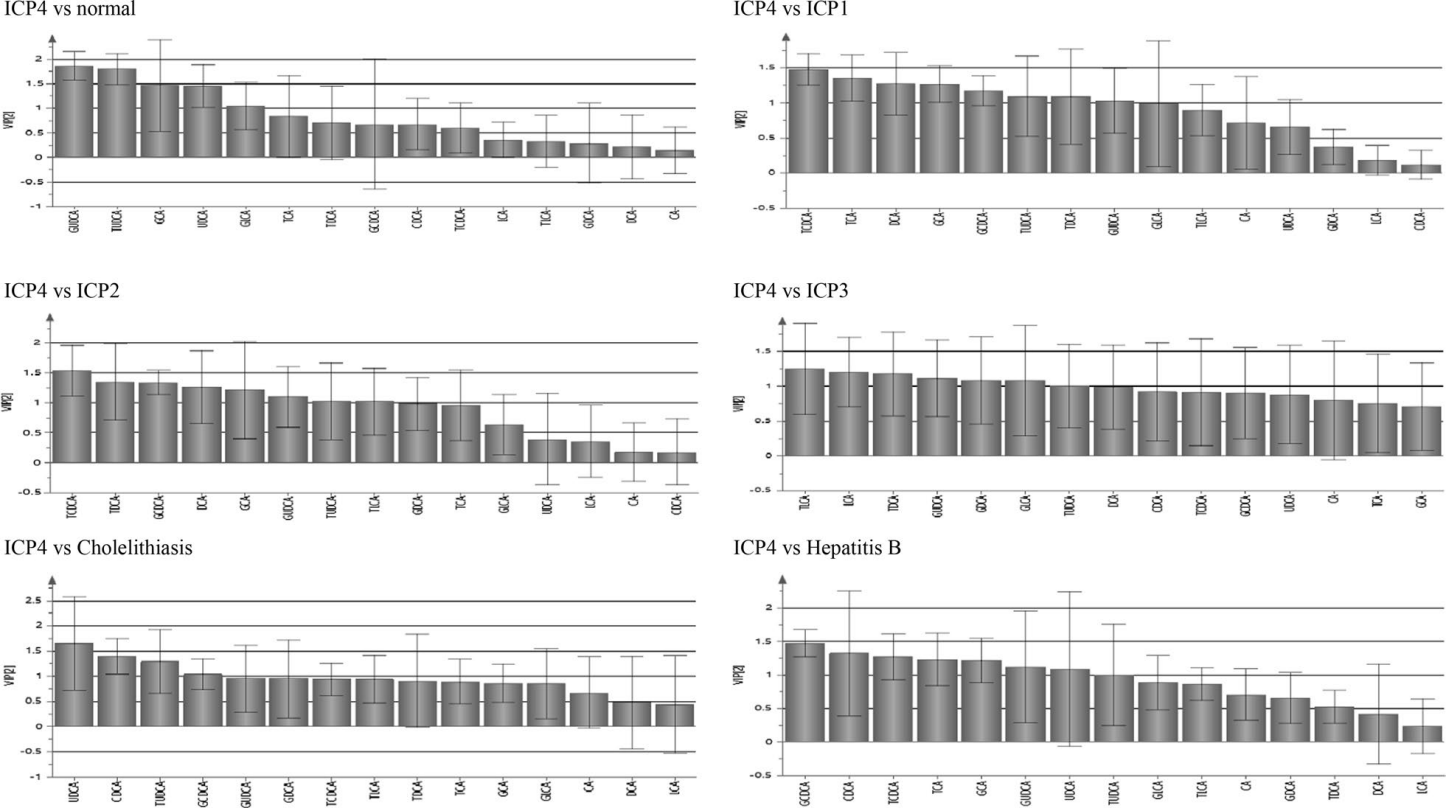
PLS-DA analysis of serum bile acid profiles VIP value between groups (ICP4 vs normal group, ICP1, ICP2, ICP3, Cholelithiasis and Hepatitis B)

The differential bile acids between ICP4 group and normal pregnant group were GCA, UDCA, GUDCA, TUDCA and GLCA, and the concentration of these bile acids in ICP4 group were significantly higher than normal pregnant group (P < 0.05). The differential bile acids between ICP4 group and ICP1 group were TCDCA, TCA, DCA, GCA, GCDCA, TUDCA, TDCA, and GUDCA. The concentration of TCDCA, TCA, GCA, GCDCA, TUDCA, TDCA, and GUDCA in ICP4 group were lower than ICP1 group (P < 0.05), while the concentration of DCA in ICP4 group was higher than ICP1 group (P < 0.05). The differential bile acids between ICP4 group and ICP2 group were TCDCA, TDCA, GCDCA, DCA, GCA, GUDCA, TUDCA, and TLCA. The concentration of TCDCA, TDCA, GCDCA, GCA, GUDCA, TUDCA, and TLCA in ICP4 group were lower than ICP2 group, while the concentration of DCA in ICP4 group was higher than ICP2 group (P < 0.05). The differential bile acids between ICP4 group and ICP3 group were TLCA, LCA, TDCA, GUDCA, GDCA, GLCA, and TUDCA, and the concentration of these bile acids in ICP4 group were lower than ICP3 group (P < 0.05). The differential bile acids between ICP4 group and cholelithiasis group were UDCA, CDCA, TUDCA, and GCDCA. The concentration of UDCA and TUDCA in ICP4 group were higher than cholelithiasis group, while the concentration of CDCA and GCDCA were lower than cholelithiasis group (P < 0.05). The differential bile acids between ICP4 group and hepatitis B group were GCDCA, CDCA, TCDCA, TCA, GCA, GUDCA, UDCA, and TUDCA, and the concentration of these bile acids in ICP4 group were lower than hepatitis B group (P < 0.05) (see Fig. 5, Table 7).

**Table7:** Analysis of Bile acids profiles between ICP4 and other groups

Comparison groups	Bile acids profile					Comparison results of bile acids concentration between groups
	CA GCA TCA	CDCA GCDCA TCDCA	DCA GDCA TDCA	LCA GLCA TLCA	UDCA GUDCA TUDCA	
ICP4 vs normal group	GCA			GLCA	UDCA GUDCA TUDCA	ICP4 > normal group
ICP4 vs ICP1	GCA TCA	GCDCA TCDCA	TDCA		GUDCA TUDCA	ICP4 < ICP1
			DCA			ICP4 > ICP1
ICP4 vs ICP2	GCA	GCDCA TCDCA	TDCA	TLCA	GUDCA TUDCA	ICP4 < ICP2
			DCA			ICP4 > ICP2
ICP4 vs ICP3			GDCA TDCA	LCA GLCA TLCA	GUDCA TUDCA	ICP4 < ICP3
ICP4 vs Cholelithiasis					UDCA TUDCA	ICP4 > Cholelithiasis
		CDCA GCDCA				ICP4 < Cholelithiasis
ICP4 vs Hepatitis B	GCA TCA	CDCA GCDCA TCDCA			UDCA GUDCA TUDCA	ICP4 < Hepatitis B

### Analysis of pregnancy outcomes in ICP subgroups

The maternal TBA level of ICP subgroups (ICP1, ICP2 and ICP3) were significantly higher than normal pregnant group (P < 0.05). The TBA level in ICP4 group was at normal level, but the concentration of UDCA, GUDCA, TUDCA, GCA and GLCA in ICP4 group were significantly higher than normal pregnant group (P < 0.01). The occurence of meconium-stained amniotic fluid, preterm delivery, and cesarean section in ICP1 group were significantly higher than ICP2 group, ICP3 group and ICP4 group, respectively (P < 0.05). The occurence of meconium-stained amniotic fluid, preterm delivery, and cesarean section in ICP2 group, ICP3 group and ICP4 group were significantly higher than normal pregnant group (P < 0.05), and no statistical differences were found among ICP2 group, ICP3 group and ICP4 group (P > 0.05) (see Table 8).

**Table 8:** Clinical information and liver function test results of normal pregnant women and patients in every subgroup of ICP

Subjects	Normal pregnant (n = 50)	ICP1 (n = 51)	ICP2 (n = 27)	ICP3 (n = 22)	ICP4 (n = 44)	H/χ^2^	P
Age (year)	28 (26, 32)	30 (27, 31)	29 (23, 33)	28 (27, 31)	29 (27, 33)	1.441	0.837
Gravidity	2 (1, 3)	2 (1, 3)	2 (1, 3)	1 (1, 2)	2 (1, 2)	8.069	0.089
Delivery pregnancy week	39 (38, 40)	37 (35, 39)^a^	37 (36, 38)^a^	38 (37, 39.0)^a^	37 (36, 39)^a^	36.587	0.000
Previous history of ICP (n, %)	0 (0%)	2 (3.9%)	5 (18.5%)^a^	2 (9.1%)	4 (9.1%)	10.855	0.028
Pregnancy outcome (n, %)							
Cesarean section	4 (8%)	42 (82.4%)^a^	18 (66.7%)^a,b^	8 (36.7%)^b^	18 (40.9%)^a,b^	53.982	0.000
Premature (< 37w)	0 (0%)	39 (76.5%)^a^	2 (7.4%)^a,b^	2 (9.1%)^a,b^	5 (11.4%)^a,b^	30.81	0.000
Meconium-stained amniotic fluid	1 (2%)	19 (37.3%)^a^	4 (14.8%)^a,b^	3 (13.6%)^a,b^	8 (18.2%)^a,b^	27.324	0.000
Neonatal ICU hospitalization rate	1 (2%)	17 (33.3%)^a^	5 (18.5%)^a^	0 (0%)	5 (11.4%)	25.422	0.000
1 min Apgar scores ≤ 7	1 (2%)	12 (23.5%)^a^	2 (7.4%)	2 (9.1%)	3 (6.8%)^b^	21.185	0.000
Newborn weight (g)	3305 (2980, 3592)	3180 (2720, 3370)^a^	2900 (2720, 3360)^a^	3185 (2837, 3320)	2960 (2565, 3870)	10.774	0.029
Postpartum hemorrhage (ml)	225 (200, 300)	200 (200, 300)	300 (200, 300)	200 (200, 285)	300 (200, 300)^a^	11.077	0.026
Liver function test results							
TBA (umol/L)	3.3 (2.4, 4.5)	32.0 (16.5, 48.1)^a^	18.8 (12.0, 28.8)^a,b^	19.9 (16.9, 29.4)^a^	6.2 (4.4, 7.4)^a,b,c^	149.387	0.000
TBIL (umol/L)	11.0 (8.8, 12.9)	18.0 (13.4, 26.6)^a^	8.4 (5.4, 12.1)^a,b^	6.5 (4.5, 10.1)^a,b^	8.2 (6.3, 10.4)^a,b^	94.044	0.000
DBIL (umol/L)	3.4 (2.6, 4.8)	12.6 (8.3, 16.8)^a^	3.7 (2.2, 5.1)^a,b^	3 (2.4, 3.6)^a,b^	3.8 (3.0, 4.5)^a,b,d^	123.664	0.000
ALT (U/L)	19.0 (17.0, 25.3)	216.0 (106.0, 336.0)^a^	176.0 (95.0, 309.0)^a^	10.0 (7.8, 13.5)^a,b^	104.5 (70.0, 174.8)^a,b,d^	142.102	0.000
AST (U/L)	18.0 (15.8, 22.0)	146.0 (73.0, 205.0)^a^	116.8 (60.0, 232.0)^a^	15.5 (12.0, 18.0)^a,b^	51.5 (35.3, 76.5)^a,b,d^	141.737	0.000

The data in the table is expressed by M (Q1, Q3)

^a^P < 0.05 compared with normal pregnant women

^b^Compared with ICP1 group, P < 0.05

^c^Compared with the ICP2 group, P < 0.05

^d^Comparing the ICP3 group with the ICP4 group, P < 0.05

## Discussion

According to the international definition of ICP, as a pregnancy-specific disease, the exclusion of abnormal liver function or elevated TBA level caused by other diseases is necessary for the clinical diagnosis of ICP. This study showed that ICP pregnant women followed this definition could actually be divided into four subtypes, and their clinical characteristics and perinatal outcomes were different. The typical clinical features of ICP usually manifested as the simultaneous elevation of TBA and ALT (ICP2 group). In addition, there are three subgroups of ICP (ICP1, ICP3 and ICP4) beside ICP2 group according to clinical practice, and this may result in confusion for obstetrican and misdiagnosis and even adverse perinatal outcomes including unnecessary iatrogenic preterm labor, cesarean section, and unexpected intrauterine fetal death. The major characteristics of ICP1 group was jaundice, that is, the serum direct bilirubin (DBIL) level was increased simultaneously with elevated TBA and ALT. In ICP3 group, maternal TBA level was significantly increased, but ALT, AST, and DBIL level were all normal; In ICP4 group, maternal ALT level was increased, but TBA and DBIL level were normal. This study analyzed the bile acid profiles of the four subgroups of ICP and found out their unique bile acid spectrum. Many studies [6, 7] have shown that ICP pregnant women and some hepatobiliary diseases may also have elevated total bile acid (TBA) level, but their bile acid profiles are different. Therefore, it has obvious limitation for the diagnosis and differential diagnosis of ICP by only use of the TBA, and we believe that the bile acid spectrum analysis is more valuable than TBA test only. The objective of this study is to investigate the characteristics of serum bile acid spectrum of four ICP subgroups, with the normal pregnant women group as negative control, and the cholelithiasis group and hepatitis B group as positive control.

### Analysis of serum bile acid profiles of ICP group

Bile acids are divided into primary and secondary bile acids. Primary bile acids are converted from cholesterol in the liver, and through a series of reactions, CA and CDCA are formed, which are conjugated with glycine and taurine, including GCA, GCDCA, TCA, and TDCA. Bile acids can be divided into hydrophilic bile acids and hydrophobic bile acids. Hydrophobic bile acids plays a role of scavenger to lead to hepatocyte necrosis by dissolving cell membrane lipids and causing increased cell membrane permeability. Hydrophilic bile acid (such as UDCA) can promote the metabolism of hydrophobic bile acids and change the composition of bile salts by increasing the content of bile acids and phospholipids in the bile, thereby protect liver cell membranes and choleretic effects against the toxicity of hydrophobic bile acids. The common types of free and conjugated bile acids in the human body are in the order of hydrophilicity: UDCA > CA > CDCA > DCA > LCA, taurine-conjugated bile acid > glycine-conjugated bile acid > free bile acid [8].

From results of our study, the characteristic bile acid profiles of ICP are CDCA, UDCA, GLCA, GCDCA, GCA, GUDCA, TCDCA, TCA, and TUDCA. The bile acid profile analysis are very helpful for clinical diagnosis of ICP and differential diagnosis to cholelithiasis or hepatitis B. The elevated glycine- and taurine-conjugated bile acids are the main composition of the bile acid spectrum of ICP pregnant women, ranging from mild to moderate degree of cytotoxic bile acids and non-toxic bile acids.

### ICP with jaundice

Maternal serum TBA and DBIL level in ICP1 group were significantly higher than normal pregnant women group and ICP2 group, ICP3 group and ICP4 group. Jaundice was the main feature of ICP1 group. The serum bile acid profiles of ICP1 group was significantly different from ICP2 group, ICP3 group and ICP4 group (see Table 5). The occurence of meconium-stained amniotic fluid, premature delivery, and cesarean delivery in ICP1 group were significantly higher than ICP2 group, ICP3 group, and ICP4 group, suggesting that ICP pregnant women with jaundice was probablely in more severe condition and adverse pregnancy outcomes, and this condition was worthy of being paid more attention clinically. The importance of bilirubin in ICP has also been noticed by many other researchers. Brouwers et al. [9] demonstrated a correlation between adverse pregnancy outcomes and liver function test in ICP. From the article by Ovadia et al. [10], stillbirth is increased when maternal TBA level is 100 μmol/L or more in ICP. It is not sufficient to propose ICP patients with bile acid level less than 100 μmol/L an expectant management based solely on bile acid level. We believe that maternal DBIL level is another important predictor of adverse pregnancy outcomes besides maternal TBA level. More research is needed to investigate the relationship between elevated maternal DBIL level and adverse perinatal outcomes to prevent from stillbirth of ICP.

### Hyperchoicemia of pregnancy

The ICP3 group (Hyperchoicemia of pregnancy) is another subtype of ICP. Maternal serum TBA level in ICP3 group is significantly increased, but the ALT level is normal, and the characteristic bile acid spectrum in ICP3 group is LCA, DCA, CDCA, UDCA, GLCA, GDCA, GCDCA, TDCA, TDCA, TCA and TUDCA. Many studies found that high TBA level in pregnant women with ICP is associated with an increased risk of severe fetal adverse events, such as spontaneous premature and iatrogenic preterm birth, fetal distress and intrauterine death [11, 12]. Our study found that maternal TBA level in ICP1 group (ICP with jaundice) was significantly higher than other ICP subgroups, and the occurrence of meconium-stained amniotic fluid, premature delivery, and cesarean delivery were significantly higher than those without jaundice ( ICP2 group, ICP3 group and ICP4 group). This result is consistent with our previous report [13, 14].

### Idiopathic aminotransferase abnormality during pregnancy

The ICP4 group (Idiopathic elevated liver enzymes during pregnancy) is also one subtype of ICP. Maternal serum TBA level in ICP4 group is normal, but ALT is elevated. Many obstetricians are confused to make diagnosis of this kind of situation. Whether this group of pregnant women is or not ICP? It is really an interesting question. In this study, we found that the characteristic bile acid profile in ICP4 group is elevated level of CDCA, UDCA, GLCA, GCDCA, GCA, GUDCA and TUDCA. Abnormal liver function diseases including viral hepatitis (hepatitis A, B, C, D, E virus, EB virus, cytomegalovirus, etc.), liver and gallstones, acute fatty liver during pregnancy, preeclampsia, gestational diabetes mellitus, autoimmune liver disease, drug-induced liver injury, and other medical complications were all excluded in ICP4 group, and maternal elevated serum ALT can not be explained by any other reason. We also found that some pregnant women in the ICP4 group had elevated serum TBA level after follow-up during pregnancy, and became typical type of biochemical characteristics of ICP.

In this study, the different bile acid profile characteristics of pregnant women in each group were obtained based on the analysis of the PLS-DA discriminant model and the VIP value through the analysis of related software. The real bile acids concentration of each group can be seen at Table 3. This study is the first to illustrate the bile acid profile characteristics of each subgroup of ICP pregnant women. The clinical biochemical characteristics (TBA, DBIL and ALT) of the ICP1 group and ICP2 group are very typical, and it is easy to diagnose based on these three biochemical indicators. The dynamic changes of the serum bile acid profile of pregnant women help to evaluate the treatment effect of ICP. Comparing the ICP1 group with the normal pregnant women group, the concentrations of toxic bile acids (GCA, TCA, DCA, GCDCA, TCDCA) and non-toxic bile acids (GUDCA, TUDCA) were significantly higher than those of normal pregnant women. In addition to the decrease of clinical biochemical indicators (TBA, DBIL and ALT), the clinical treatment effect also needs to pay attention to the decrease of toxic bile acid (GCA, TCA, DCA, GCDCA, TCDCA) concentration in the blood of pregnant women in the ICP1 group. The clinical biochemical characteristics of ICP3 group and ICP4 group are quite special. The ICP3 group mainly showed elevated TBA levels, while DBIL and ALT were normal. Comparing the ICP3 group with normal pregnant women, the concentration of toxic bile acids (LCA, GLCA, TLCA, CDCA) and non-toxic bile acids (UDCA, GUDCA, TUDCA) was significantly higher than that of normal pregnant women. The clinical treatment effect is not only concerned with the decrease of pregnant women's serum TBA levels. It is also necessary to pay attention to the decrease in the concentration of toxic bile acids (LCA, GLCA, TLCA, CDCA) in the blood of pregnant women in the ICP3 group. The ICP4 group mainly showed elevated ALT levels, while TBA and DBIL were normal. Comparing the ICP4 group with the normal pregnant women group, the concentrations of toxic bile acids (GCA, GLCA) and non-toxic bile acids (UDCA, GUDCA, TUDCA) were significantly higher than those of normal pregnant women. In addition to paying attention to the serum ALT level of pregnant women, the clinical treatment effect also needs to pay attention to the decrease of toxic bile acid (GCA, GLCA) concentration in the blood of pregnant women in the ICP4 group.

Ursodeoxycholic acid (UDCA) is currently the first-line drug for the treatment of ICP. However, Chappell et al. [15] found that although UDCA can reduce the serum levels of TBA and ALT in pregnant women with ICP, it did not significantly reduce the risk of adverse fetal outcomes (stillbirth and/or premature delivery), the cause of which is unclear. According to the results of this study, the analysis of serum bile acid profiles of ICP pregnant women is helpful to judge the effect of UDCA treatment, and at the same time, it helps to explore more ICP treatment drugs to reduce the serum toxic bile acid levels of pregnant women and avoid adverse pregnancy outcomes.

## Conclusions

In summary, this study found that there are four subtypes of ICP pregnant women, and bile acid spectrum analysis is helpful for diagnosis and differential diagnosis. ICP with jaundice could be an important predictor of adverse pregnancy outcomes of ICP and should be paid more attention in clinical practice. Hyperchoicemia of pregnancy and idiopathic aminotransferase abnormality are also subtypes of ICP, and need to be carefully followed up.

## Supplementary Information

**Additional file 1.** ICP1: original data.

**Additional file 2.** ICP2: original data.

**Additional file 3.** ICP3: original data.

**Additional file 4.** ICP4: original data.

**Additional file 5.** Cholelithiasis: original data.

**Additional file 6.** Hepatitis B virus: original data

**Additional file 7.** Normal pregnant women: original data

## Data Availability

All data generated or analysed during this study are included in this published article.

## Abbreviations

UPLC-MS/MS: Ultra performance liquid chromatography-mass spectrometry/mass spectrometry
ICP: Intrahepatic cholestasis of pregnancy
HP: Hyperchoicemia of pregnancy
TBA: Total bile acid
CA: Cholic acid
CDCA: Chenodeoxycholic acid
UDCA: Ursodeoxycholic acid
LCA: Lithocholic acid
DCA: Deoxycholic acid
GCA: Glychocholic acid
GCDCA: Glychochenodeoxycholic acid
GDCA: Glychodeoxycholic acid
GLCA: Glycholithocholic acid
GUDCA: Glychoursodeoxycholic acid
TCA: Taurocholic acid
TCDCA: Taurochenodeoxycholic acid
TUDCA: Tauroursodeoxycholic acid
TDCA: Taurodeoxycholic acid
TLCA: Taurolithocholic acid
TBIL: Total bilirubin
DBIL: Conjugated bilirubin
ALT: Alanine aminotransferase
AST: Aspartate aminotransferase
